# Wide-narrow row spacing reduces mechanical harvest losses in rice by optimizing panicle distribution: evidence from five-year field trials integrating density and cultivar variables

**DOI:** 10.3389/fpls.2026.1796989

**Published:** 2026-04-17

**Authors:** Yuedong Li, Maoxing Song, Yue Zhang, Nan Xiao, Tiexin Yang, Liang Ma, Liqiang Dong, Shuang Wang

**Affiliations:** 1 Liaonning Rice Research Institute, Liaoning Academy of Agricultural Sciences, Shenyang, China; 2 Tangshan Academy of Agricultural Sciences, Tangshan, Hebei, China; 3 Jilin Academy of Agricultural Sciences (Jilin Agricultural Products Quality Supervision and Inspection Center), Jilin, China; 4 Dengta Agricultural and Rural Affairs Service Center, Liaoyang, Liaoning, China

**Keywords:** integration of agronomic and agricultural machinery, panicle distribution, rice yield, row spacing method, yield loss

## Abstract

**Objective:**

The aim of this study was to establish a mechanized rice production technique that simultaneously increases grain yield and reduces harvest loss.

**Methods:**

Large-scale field trials were conducted comparing two planting methods, the traditional farmers’ equal−row spacing method (FM) and the wide−narrow row spacing method (WNM), and investigated their effects on yield, yield components, plant uniformity at maturity, mechanical harvest loss, and varietal performance.

**Results:**

The results demonstrate that WNM enhances yield through two complementary mechanisms. First, it significantly increases the number of productive panicles per unit area, with the high−yield cultivar Lijiang 419 exhibiting a greater response (average yield increasing of 5.5%). Second, WNM markedly reduces mechanical harvest loss by decreasing panicle dropping. The wider row spacing of WNM (36 cm vs. 30 cm in FM) provides larger corridors for the combine header, minimizing incidental contact with adjacent plants and thereby reducing dropped panicles. Additionally, WNM improves panicle neck height uniformity by 4.7–5.0%, ensuring that more panicles are positioned above the cutting height and further mitigating losses.

**Conclusion:**

Consequently, the integration of optimized agronomic practices (WNM) with suitable machinery effectively boosts both yield and harvest efficiency, making WNM a promising planting pattern for mechanized rice production areas seeking high yield and low loss.

## 1 Introduction

As multi-output crop, rice (*Oryza sativa* L.) supporting global food security (Jayaprakash et al., 2022; Yuan et al., 2021; Tonda et al., 2024.). Rice occupies 165 million hectares worldwide, with China contributing critically to both cultivation area (18%) and total output (27%) (FAO-STAT, 2022). Within China, Liaoning Province emerges as a key japonica rice production base, characterized by high mechanization adoption and yield potential (Dong et al., 2023; Li et al., 2023). The ongoing rural labor transition necessitates mechanized solutions across the production chain (Zhu et al., 2019; Jiang et al., 2024). However, current machine-transplanted rice systems predominantly employ uniform 30-cm row spacing (FM), which imposes critical constraints: Limited spatial optimization: Restricts light penetration and tiller development (Zhang et al., 2020). Harvest inefficiencies: Induces panicle height heterogeneity (>75 cm panicles vs. short tillers), causing significant harvest losses through header contact and dropped panicles (Wang et al., 2022). While yield enhancement typically focuses on genetic improvement or input management (e.g., fertilization, water control; Zhang et al., 2022), structural optimization of crop populations remains underexplored.

The structural uniformity of maturity rice populations critically determines yield-forming processes (Du et al., 2014; Wang et al., 2022). Higher population homogeneity promotes synchronous tillering and increases productive panicle number per unit area. Optimized canopy architecture enhances light interception and biomass accumulation in panicles rather than vegetative organs. Consequently, population quality directly governs both yield potential and mechanical harvest efficacy through its effects on panicle distribution. Wide narrow row planting technology is an innovative technology that has emerged in recent years, attracting more and more attention (Tang et al., 2025). With the improvement of supporting agricultural machinery technology, wide narrow row planting technology has successfully achieved mechanized transplanting in crops such as corn, rice, soybeans, and cotton. The implementation of mechanized rice cultivation operations with alternating wide–narrow row spacings improves the light, temperature, water, and heat conditions of the rice growth environment through the optimization of the spacings between plants and rows, the establishment of a suitable population structure, and the coordination of the relationships between plant populations and individuals, thereby maximizing the potential for increased yields of rice cultivars (Wang et al., 2015; Zhu et al., 2018; Hu et al., 2020; Dong et al., 2023). By providing ventilated and transparent corridors in wide rows while changing the horizontal light conditions into three-dimensional light conditions, the marginal advantage of the population is fully utilized, which can potentially increase cultivar yields (Hu et al., 2018; Xiong et al., 2022). Hu et al. (2020) found that the wide and narrow row pattern increased rice yield by 8-12% by increasing the number of spikelets per panicle.

An important measure for achieving cost savings and high efficiency in mechanized rice harvesting (Alizadeh and Allameh, 2013). At present, rice harvesting in China is achieved mainly through semi-fed and fully fed harvesters (Yang et al., 2014; Ding et al., 2022). In the operation of semi-fed harvesters, plants are cut, panicles are threshed in the threshing bin, and straw is removed. In contrast, in the operation of fully fed harvesters, entire plants are cut and transported to the threshing bin for threshing, after which straw is removed. Between these methods, the semi-fed harvesting method aims to retain and fully utilize all of the cut straw, positioning it as the main harvesting method in the region. The semi-fed harvester serves as the predominant harvesting method in our region due to its capacity to preserve rice straw integrity. However, there are still differences in mechanized harvesting losses due to variety, transplanting density, and transplanting methods in production practice. According to government statistics, 11.76 billion kg of rice is lost annually, with average loss rates ranging from 5.17% to 11.98%. The mechanical harvesting quality of existing rice production methods is relatively low, and the degree of harvesting loss is relatively high. In production, there is an urgent need for techniques to help reduce rice yield losses during mechanical harvesting (Luo et al., 2020; Xu, 2021; Xu et al., 2023). Faced with the requirements of efficient and high-quality agricultural development after mechanization, increasing yields while reducing harvest losses is an inevitable trend in rice production in this region (Li et al., 2020; Yu et al., 2021; Zhao et al., 2023). Therefore, because improving the plant and panicle morphological characteristics at the mature stage of panicle height uniformity and minimizing unharvested short panicles is an effective way to enhance the quality of harvested rice, increasing the quality of mechanized harvesting has become an important issue in the development of regional rice mechanization.

The effects of different transplanting methods on the quality of mechanically planted rice populations remain inconsistent, and how these effects interact with cultivar selection and inter-annual environmental variation is poorly understood. Furthermore, limited research has addressed the integration of planting methods and cultivars to optimize mechanized harvest quality. To address these gaps, we conducted large-scale mechanized field experiments over five consecutive years in the central plain of Liaoning Province. The specific objectives of this study were to: (1) evaluate the effects of different planting methods (traditional equal-row spacing vs. wide-narrow row spacing) and cultivars on rice yield and its components; (2) quantify mechanical harvest losses and identify their determinants under different planting configurations; (3) elucidate the mechanisms by which planting method and cultivar influence productive panicle number and panicle position uniformity at maturity; and (4) identify optimal combinations of planting method and cultivar that achieve high and stable yields while minimizing harvest losses, thereby supporting the integration of agronomic practices with mechanized production systems. Clarifying these relationships will provide farmers with evidence-based strategies to reduce yield loss risks and achieve sustainable, high-yield rice production.

## 2 Materials and methods

### 2.1 Experimental design

A field experiment was conducted from 2019 to 2023, with the traditional farmers’ equal-row spacing (traditional mechanization) method (FM) and the wide–narrow row spacing (optimized mechanization) method (WNM) employed for each of the two cultivars considered, resulting in the following experimental groups: LJFM (Liaojing 419 with the FM), LJWNM (Liaojing 419 with the WNM), LYFM (Tianlongyou 619 with the FM), and LYWNM (Tianlongyou 619 with the WNM). Seedlings were obtained through industrialized seedling production. Each seedling tray was 580 mm long, 280 mm wide, and 30 mm deep, with a sowing rate of 3800 seeds. During transplantation, the transplanter carried seedlings horizontally 18 times and planted them vertically to a depth of 12 mm, with 3~5 seedlings (average 4seedlings/hill) carried each time. FM was limited to fixed equal-row spacing (row spacing: 30 cm; hill spacing: 18 cm), a Kubota transplanter 2ZGQ-6D1 (SPV-6CMD, Kubota, Suzhou, China) was adopted. WNM was specifically designed to accommodate the wide–narrow row spacing configuration (wide-row spacing: 36 cm; narrow-row spacing: 14 cm), a Jiufu rice transplanter 2ZG-8DKZ (G8KZ, Jiufu, Suzhou, China) was used. Both transplanters were equipped with the same intelligent navigation system (Shanghai Lianshi Navigation Technology Co., Ltd.) to ensure consistent operational precision. Both machines were operated by experienced technicians, and visual inspections confirmed comparable transplanting quality (seedling depth consistency, missing hill rate, and seedling injury) across treatments. A randomized complete block design with three replications was employed. Each treatment plot measured 1000 m² (40 rows × 100 m length). The experiment was conducted at the same field site over five consecutive years (2019–2023). To account for potential spatial heterogeneity, soil samples were collected from each block prior to the first season and analyzed for baseline properties (pH, organic matter, available N, P, K), which showed no significant differences among blocks. Blocks were re-randomized annually to avoid confounding temporal trends with spatial effects.

### 2.2 Cultivars

Liaojing 419 (LJ419), provided by the Liaoning Rice Research Institute, is a conventional japonica rice cultivar that exhibits intermediate to late maturation. It provides an average yield of 10380 kg/ha, an average plant height of 104.0 cm, an average panicle length (PL) of 16.2 cm, an average number of grains per panicle of 128.7, a seed setting rate of 87% and an average thousand-grain weight of 24.5 g. Liaojing 419 is a regionally recognized high-yield cultivar selected for its mechanization adaptability in Liaoning Province, China.

Tianlongyou 619 (LY619), provided by Tianjin Tianlong Seed Technology Co., Ltd., is a hybrid japonica rice cultivar that is among the cultivars in Liaoning Province with early maturation, high quality, and flavorful grains. The average yield is 9500 kg/ha, the average plant height is 109.5 cm, the average panicle length (PL) is 17.6 cm, the average number of grains per panicle is 111.2, a seed setting rate of 85% and the average thousand-grain weight is 27.8 g. Tianlongyou 619 cultivar is a good-tasting cultivar that is widely cultivated in northern China.

### 2.3 Experimental site

**Figure 1:**
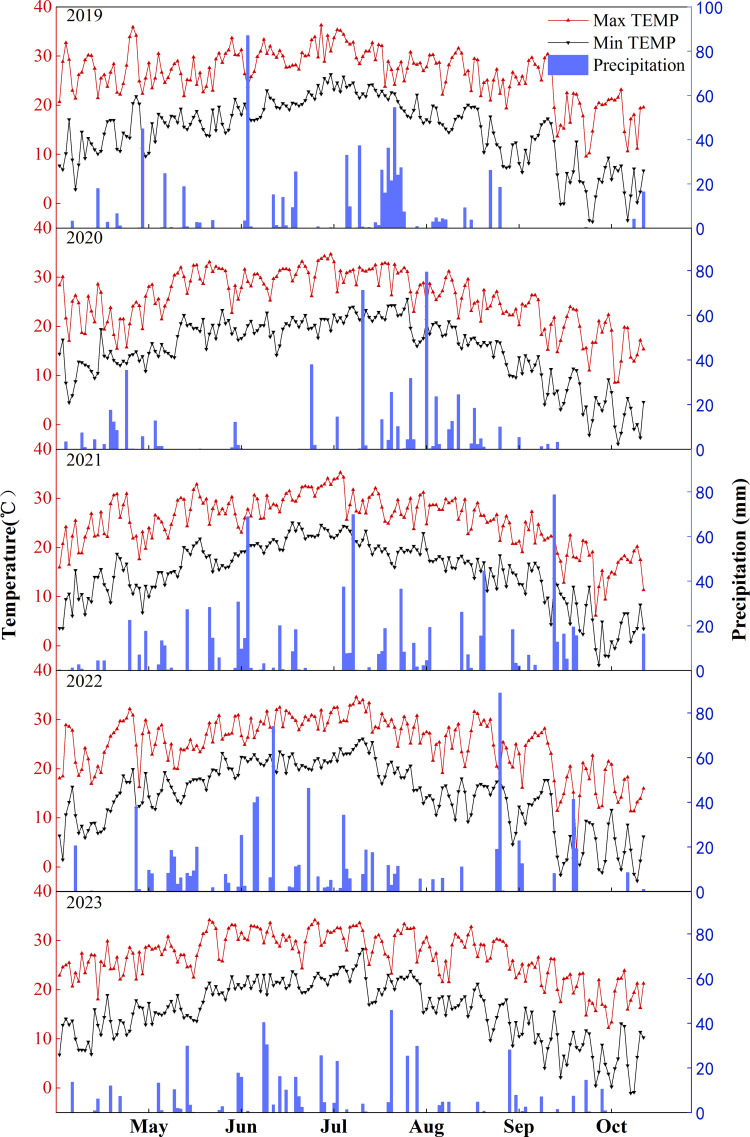
Meteorological data for the rice growth period at the study site from 2019 to 2023.

The field experiment was conducted at a representative large-scale and highly mechanized rice production base in the central plain of Liaoning Province—the Liaoning Rice Research Institute Experimental Base in Dengta city (123.18°E, 41.49°N). This site is located in the Northeast China Plain, a major japonica rice production region with a warm temperate monsoon climate. The base features advanced production techniques, standardized field management, and mature mechanized cultivation systems, making it highly representative of the local rice farming practices and providing findings with strong potential for replicability and extension. The seasonal average precipitation and maximum temperature are 701.4 mm and 25.9 °C, respectively. Meteorological data for the rice-growing seasons are shown in **Figure 1**.

The initial soil pH in the study area (spring 2019) was 6.2, with an organic matter content of 2.63%. The levels of available nitrogen, available phosphorus, and available potassium were 89.3, 46.4, and 83.9 mg/kg, respectively. In this study, the fertilizer application rate was determined on the basis of the characteristics of the two cultivars. Notably, a higher fertilizer application rate was employed for the high-yield cultivar LJ419 than for the good-tasting variety LY619. LJ419 and LY619 were planted in adjacent but separated blocks within the same paddy field, each plot was isolated by 50-cm-high reinforced soil bunds (width: 30 cm) with plastic membrane cores (0.3 mm thickness) to prevent water/nutrient exchange.

For the high-yield cultivar LJ419, the local high-yield cultivation fertilization method was adopted. The application rate of pure N (slow-release urea) for LJ419 was 300.0 kg/ha, and the basal–tillering–panicle fertilizer ratio was 6:3:1. The application rate of pure P (diammonium phosphate) was 69.0 kg/ha, with all base fertilizers applied. The application rate of pure K (potassium chloride) was 75.0 kg/ha, and potassium, base and panicle fertilizers were applied in equal amounts. Mechanized transplanting was implemented on May 13, 2019, May 13, 2020, May 14, 2021, May 15, 2022, and May 13, 2023, and mechanized harvesting was conducted on October 8, 2019, October 4, 2020, October 14, 2021, October 6, 2022, and October 9, 2023, respectively.

For the good-tasting cultivar LY619, the local high-quality high-taste cultivation fertilization method and transplanting date was adopted. The application rate of pure N (slow-release urea) for LY619 was 150.0 kg/ha, which was applied at a basal–tillering fertilizer ratio of 6:4. The application rate of pure P (diammonium phosphate) was 69.0 kg/ha, with all base fertilizers applied. The application rate of pure K (potassium chloride) was 75.0 kg/ha, and all base fertilizers were applied. Mechanized transplanting was conducted on May 18, 2019, May 18, 2020, May 20, 2021, May 21, 2022, and May 19, 2023, and mechanized harvesting occurred on October 8, 2019, October 4, 2020, October 14, 2021, October 6, 2022, and October 9, 2023, respectively.

The field was maintained with a thin water layer (3–5 cm) during mechanical transplanting and irrigated with a stable shallow water layer at a depth of approximately 2–3 cm at the tillering stage. When the number of tillers reached 80% of the expected number of panicles, drainage and field setting began. From the jointing stage to the mature stage, alternate wetting and moderate soil drying irrigation management was employed until 10–12 d before the harvest. Other management plans during the reproductive period were implemented according to Dong et al. (2023).

### 2.4 Measurement methods

#### 2.4.1 Growth dynamics

Stem and tiller dynamics were assessed as follows: Consecutive 20 hills were marked per plot at transplanting, stem and tiller count survey was conducted every 7 d from transplanting to the full heading stage of rice plants, and the maximum number of tillers was determined on the basis of tiller dynamics. For clarity and to highlight key transitional periods, data from five representative growth stages (transplanting, initial tillering, peak tillering, heading, and maturity) are presented in Figure.

Tiller counting: All stems >3 cm height within quadrats were tallied.
Maximum tiller determination: Peak value recorded when three consecutive counts showed ≤ 3% change.

#### 2.4.2 Actual yield

After the grain-ripening period, a Kubota 4LBZ-172B (PRO888GM) semifed harvester (with a header width of 1720 mm and an average cutting height of 15 ± 1 cm above the ground, Threshing drum speed 550 rpm, Ground speed: 0.8 m/s) was employed for mechanized harvesting. The yield was calculated through conversion on the basis of a 14.5% moisture content.

#### 2.4.3 Grain yield composition

The factors contributing to the rice yield are as follows: Before large-scale harvesting, 10 hills of plants with uniform and consistent growth were selected in each plot (2 adjacent rows with 5 consecutive holes were selected under the WNM) for indoor seed testing. The following parameters were measured: productive panicle number (PPN), PL, primary branch number (PBN), primary branch filled grain number (PBFGN), primary branch seed setting rate (PBSSR), primary branch thousand-grain weight (PBTGW), secondary branch number (SBN), secondary branch filled grain number (SBFGN), secondary branch seed setting rate (SBSSR), and secondary branch thousand-grain weight (SBTGW). The rice panicle formation rate was calculated as the proportion of productive panicles to the maximum number of tillers.

Productive panicles: Panicles with ≥20 filled grains (Dong et al., 2023).
Panicle length (PL): Base of rachis to tip.

#### 2.4.4 Panicle neck height and uniformity (U_pnh_)

Before large-scale harvesting, 10 consecutive holes of plants with uniform and consistent growth were selected in each community (2 adjacent rows with 5 consecutive holes under the WNM), and the heights of the neck nodes of all the plants in the field were measured under natural conditions. The uniformity of the panicle neck height (Upnh) was calculated on the basis of the degree of variation (Equation 1):

(1) $$U_{pnh}=[1-(S_{pnh}/\bar{X_{pnh}})]\times 100$$

Where $S_{pnh}$ the standard deviation of panicle neck height, and $\bar{X_{pnh}}$is the mean panicle neck height. $S_{pnh}$ is computed as: $S_{pnh}=\sqrt{1/(n-1)\sum_{i=1}^{n}{\left(Xi_{pnh}-\bar{X_{pnh}}\right)}^{2}}$where S_pnh_ denotes the standard deviation of the panicle neck height; Xi_pnh_ denotes the observed panicle neck height; and $\bar{X_{pnh}}$ denotes the average panicle neck height. The theoretical range of Upnh is (-∞,100]. A value of 100 indicates perfect uniformity (all plants with identical panicle neck height). As variability increases, Upnh decreases and may become negative, which would imply extremely high dispersion. In such cases, we consider values less than zero as indicating the lowest uniformity and treat them as zero for practical interpretation. This index was chosen instead of directly reporting the coefficient of variation (CV = S/ $\bar{X}$) because Upnh expresses uniformity as a percentage, with higher values directly representing greater uniformity, making it more intuitive for readers to grasp the degree of population evenness.

#### 2.4.5 Dropped panicle traits and uniformity (U_dpl_ and U_dpgn_, respectively)

During mechanized harvesting in each plot, a 1.5 m × 1.8 m area (FM: 5 rows, 10-hole distance; WNM: 6 rows, 10-hole distance) was selected, in which the harvester header remained horizontally perpendicular to the row walking area. The dropped panicle number (DPN), dropped panicle length (DPL) (including the plant part), dropped panicle grain number (DPGN), and dropped panicle grain weight (DPGW) were obtained in the field after harvesting and threshing.

The dropped panicle length uniformity (Udpl) was calculated on the basis of the degree of variation in the lengths of dropped panicles (Equation 2):

(2) $$U_{dpl}=[1-(S_{dpl}/\bar{X_{dpl}})]\times 100$$

$$S_{dpl}=\sqrt{1/(n-1)\sum_{i=1}^{n}{\left(Xi_{dpl}-\bar{X_{dpl}}\right)}^{2}}$$

where S_dpl_ denotes the standard deviation of the lengths of dropped panicles; Xi_dpl_ denotes the observed value of the DPL; and $\bar{X_{\text{dpl}}}$ denotes the average DPL.

The dropped panicle grains number uniformity (U_dpgn_) was calculated on the basis of the degree of variation (Equation 3):

(3) $$U_{dpgn}=[1-(S_{dpgn}/\bar{X_{dpgn}})]\times 100$$

$$S_{dpgn}=\sqrt{1/(n-1)\overset{n}{\underset{i=1}{\sum }}{\left(Xi_{dpgn}-\bar{X_{dpgn}}\right)}^{2}}$$

where S_dpgn_ denotes the standard deviation of the dropped panicle grains number; Xi_dpgn_ denotes the observed value of th dropped panicle grains number; and $\bar{X_{\text{dpgn}}}$ denotes the average dropped panicle grains number.

#### 2.4.6 Dropped panicle rate

The number of dropped panicles referred to in Section 2.4.5 was calculated, and the percentage of dropped panicles (DPR) was calculated as follows:


DPR=DPN/(PPN+DPN)×100

#### 2.4.7 Dropped panicle weigh

All dropped panicles, as mentioned in Section 2.4.6, were manually threshed and the dropped panicle grain weight was recorded.

#### 2.4.8 Yield loss rate

The yield loss rate was calculated as follows:


YLR=DPW/(AY+DPW)

### 2.5 Data analysis.

Excel (2020, Microsoft, Redmond, WA, USA) was used for data organization, SPSS (22.0; SPSS Inc., Chicago, IL, USA) was used for analysis of variance. Data are presented in mean form. Considering Y as a random effect, V and M as fixed effects, a linear mixed-effects model was employed to analyze their individual and interactive effects on various indicators, followed by *post-hoc* multiple comparisons using Tukey HSD. Pearson correlation was used to examine the linear relationship between each indicator and yield as well as mechanization loss. R4.5.2 (R Foundation for Statistical Computing, Vienna, Austria) and Origin (Origin Lab, Hampton, MA, USA) was used for data plotting. The Mantel test was conducted on R4.5.2 and free online platform ChiPlot (https://www.chiplot.online/) to assess the correlation between each parameter and yield.

## 3 Results

### 3.1 Mechanized-harvesting yield

**Figure 2:**
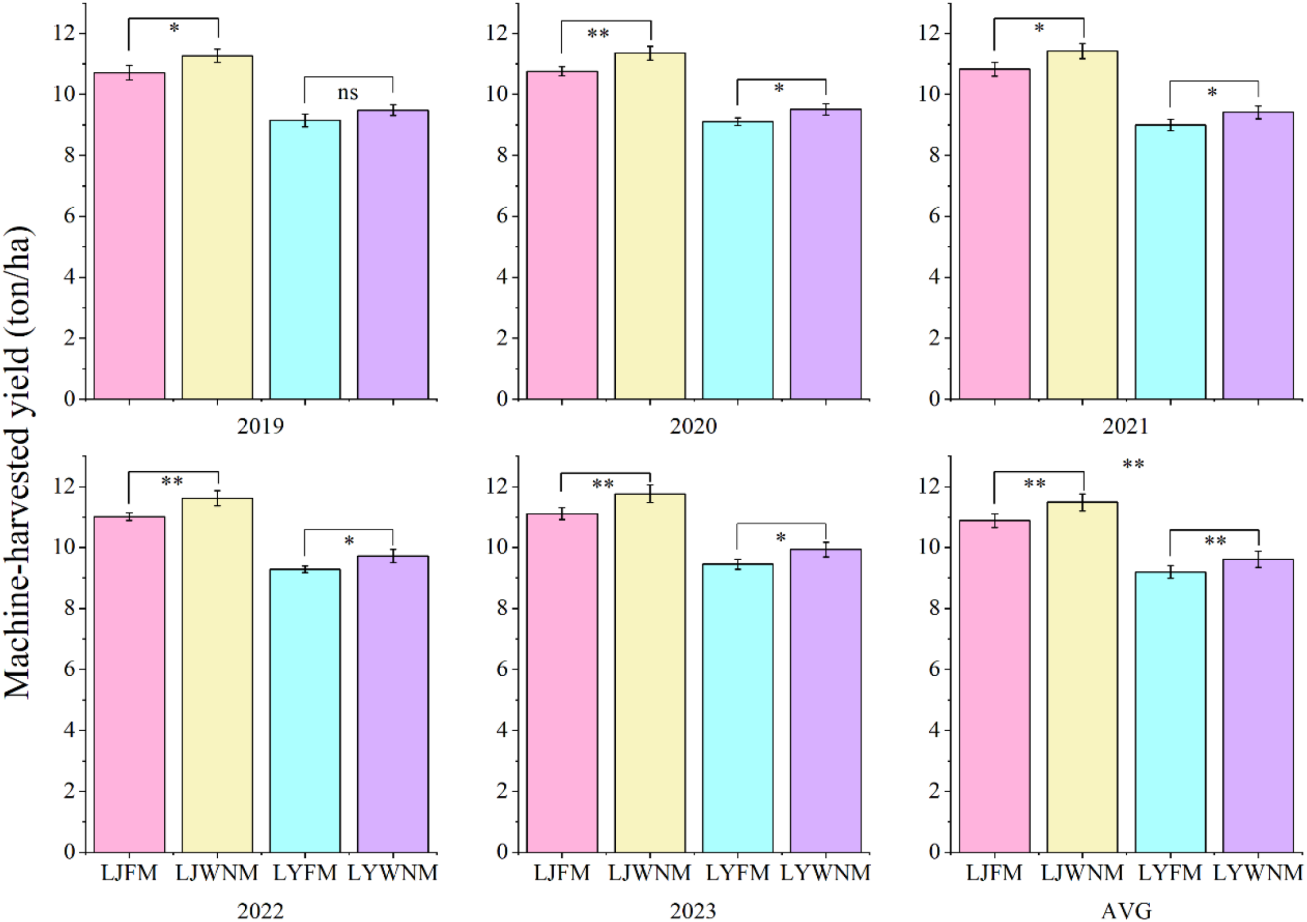
Mechanized-harvesting yield. AVG is 2019–2023 average actual yield values. LJFM is Liaojing419 with traditional farmers’ equal row spacing method, LJWNM is Liaojing419 with wide-narrow row spacing method, LYFM is Tianlongyou619 with traditional farmers’ equal row spacing method, LYWNM is Tianlongyou619 with wide-narrow row spacing method. Values within a column marked by * and ** indicate significance at the 0.05 and 0.01 levels, respectively, between different years.

The quality of harvested grain is the most important indicator of rice production. According to the analysis of the five-year actual yields of the two cultivars under the two methods (**Figure 2**), the use of the WNM significantly (p<0.05) or extremely significantly (p<0.01) enhanced the yields of the two cultivars, except for LY619 in 2019. The machine-harvested yield of the LJ419 cultivar was greater than that of the LY619 cultivar. The five-year average actual production of the LJ419 cultivar under the WNM was 0.60 tons/ha greater than that under the FM, with an average yield increase rate of 5.5%. The machine-harvested yield of the LY619 cultivar under the WNM was 0.41 tons/ha greater than that under the FM, with an average yield increase rate of 4.5%. Under the same method, the LJ419 cultivar exhibited a greater response in terms of the machine-harvested yield. Moreover, for the same cultivar, the use of the WNM was more conducive to increasing the actual yield.

### 3.2 Grain yield composition factors

The final grain yield emerges from dynamic interactions among yield components. According to the analysis of the yield components of the two cultivars under the two methods over five years (**Table 1**), there were significant differences in the PBFGN and SBFGN between methods of LJ419, whereas the differences in the other indicators between years were not significant. Overall, variety LJ419 tended to excel in yield-related traits like PPN, SBN, and SBFGN, while LY619 performed better in quality traits like PBTGW and SBGSR. Compared to FM, WNM significantly increased PPN but concurrently reduced PBFGN and SBFGN, indicating a redistribution of yield components rather than a complete elimination of the trade-off among them. However, the net effect of this redistribution was a modest but consistent increase in total grain yield (4.0–5.5%, **Figure 2**), suggesting that WNM achieves a more favorable component balance under the given ecological conditions. Year effects were limited but amplified through interactions, reflecting environmental variability. These results suggest that selecting LJ419 with WNM could maximize productivity in some contexts, while LY619 with FM might optimize grain quality, depending on the target trait and year.

**Table 1:** Grain yield composition factors.

Y	M	PPN 10^4^/ha	PL cm	PBN	PBFGN	PBGSR %	PBTGW g	SBN	SBFGN	SBGSR %	SBTGW g
2019	LJFM	351.1Bb	18.9Aa	11.9Aa	58.8Aa	95.7Aa	25.3Aa	24.6Aa	71.2Aa	81.7Aa	23.9Aa
	LJWNM	411.2Aa	17.9Ab	11.9Aa	53.1Bb	95.3Aa	25.1Aa	24.0Aa	67.4Bb	84.9Aa	23.2Aa
	LYFM	323.1Aa	19.2Aa	11.8Aa	54.3Aa	97.4Aa	28.2Aa	24.9Aa	58.9Aa	85.5Ab	24.2Aa
	LYWNM	340.0Ab	18.4Ab	11.7Aa	54.7Aa	97.8Aa	27.7Aa	24.9Aa	56.0Ab	88.9Aa	24.6Aa
2020	LJFM	355.5Bb	18.6Aa	11.7Aa	59.5Aa	95.7Aa	25.5Bb	27.0Aa	68.6Aa	81.1Ab	23.5Aa
	LJWNM	418.9Aa	18.3Aa	11.8Aa	53.8Bb	95.3Aa	25.3Bb	24.5Bb	63.2Bb	84.1Aa	23.4Aa
	LYFM	320.1Bb	19.1Aa	11.8Aa	55.6Aa	97.8Aa	28.0Aa	24.9Aa	57.2Aa	85.1Ab	24.7Aa
	LYWNM	347.4Aa	18.5Ab	11.6Aa	53.8Aa	97.1Aa	27.7Aa	24.9Aa	55.0Ab	88.7Aa	24.1Aa
2021	LJFM	352.7Bb	18.9Aa	12.0Aa	63.5Aa	96.0Aa	25.7Aa	27.8Aa	66.7Aa	80.7Aa	23.0Aa
	LJWNM	422.2Aa	18.2Aa	11.9Aa	54.5Bb	95.4Aa	25.5Aa	25.8Bb	62.2Bb	83.8Aa	23.7Aa
	LYFM	315.9Bb	19.4Aa	11.7Aa	54.0Aa	96.8Aa	28.5Aa	24.9Aa	60.5Aa	85.8Ab	24.1Aa
	LYWNM	341.8Aa	18.3Ab	11.6Aa	54.0Aa	97.4Aa	28.2Aa	24.9Aa	58.1Aa	89.3Aa	23.5Aa
2022	LJFM	357.4Bb	18.7Aa	12.1Aa	61.5Aa	95.8Aa	26.0Aa	28.4Aa	70.0Aa	81.4Ab	23.3Aa
	LJWNM	427.9Aa	18.1Aa	11.8Aa	52.1Bb	95.2Aa	25.9Aa	25.1Bb	66.2Bb	84.7Aa	23.1Aa
	LYFM	318.2Bb	18.9Aa	11.8Aa	54.2Aa	97.7Aa	29.0Aa	24.9Aa	61.6Aa	86.0Ab	23.9Aa
	LYWNM	350.3Aa	18.5Aa	11.8Aa	53.3Aa	97.4Aa	28.8Aa	24.9Aa	57.4Bb	89.2Aa	24.1Aa
2023	LJFM	357.1Bb	19.0Aa	12.1Aa	59.9Aa	95.8Aa	26.3Aa	27.2Aa	69.5Aa	81.3Ab	24.0Aa
	LJWNM	425.4Aa	18.4Aa	11.9Aa	53.2Bb	95.3Aa	26.0Aa	26.7Aa	67.1Aa	84.9Aa	23.0Ab
	LYFM	324.8Ab	19.1Aa	12.1Aa	58.8Aa	97.9Aa	28.2Aa	24.9Aa	57.9Aa	85.3Ab	24.1Aa
	LYWNM	343.8Aa	18.7Aa	11.5Ab	57.5Aa	97.6Aa	28.1Aa	24.9Aa	55.3Ab	88.8Aa	23.7Aa
F-value	V	1041.36**	81.46**	12.30*	3.33ns	2619.41**	238.17**	5.34ns	57.69**	308.47**	42.90**
	M	459.26**	45.20**	6.33ns	93.97**	164.46**	80.20**	10.21*	176.23**	3723.75**	2.58ns
	Y	1.19ns	1.02ns	1.74ns	0.60ns	1.03ns	3.58ns	1.00ns	0.68ns	0.68ns	0.00ns
	V×M	339.23*	0.18ns	0.32ns	47.50**	81.05**	3.66ns	10.20*	2.95ns	1.72ns	0.02ns
	V×Y	2.25ns	0.31ns	0.60ns	5.05ns	2.98ns	56.27**	3.32ns	13.54*	13.92*	0.26ns
	M×Y	3.41ns	2.09ns	0.92ns	0.75ns	0.52ns	1.36ns	0.99ns	0.59ns	0.66ns	0.46ns
	V×M×Y	0.37ns	0.53ns	0.97ns	2.87*	0.03ns	0.27ns	4.66**	1.13ns	0.24ns	2.95*

LJ, variety of LJ419; LY, variety of TLY619; FM, traditional farmers’ equal row spacing method; WNM, wide-narrow row spacing method; Y, year; V, variety; M, method; Y, year; ns, no significant difference; * and ** indicate significance at the 0.05 and 0.01 levels, respectively. PPN, productive panicle number; PL, panicle length; PBN, primary branch number; PBGN, primary branch filled grain number; PBSSR, primary branch seed setting rate; PBTGW, primary branch thousand-grain weight; SBN, secondary branch number; SBGN, secondary branch filled grain number; SBSSR, secondary branch seed setting rate; SBTGW, secondary branch thousand-grain weight; Values within a column followed by different uppercase letters and lowercase letters are significantly different at the 0.05 and 0.01 levels in same varieties and in one year.

### 3.3 Tiller and stem dynamics

**Figure 3:**
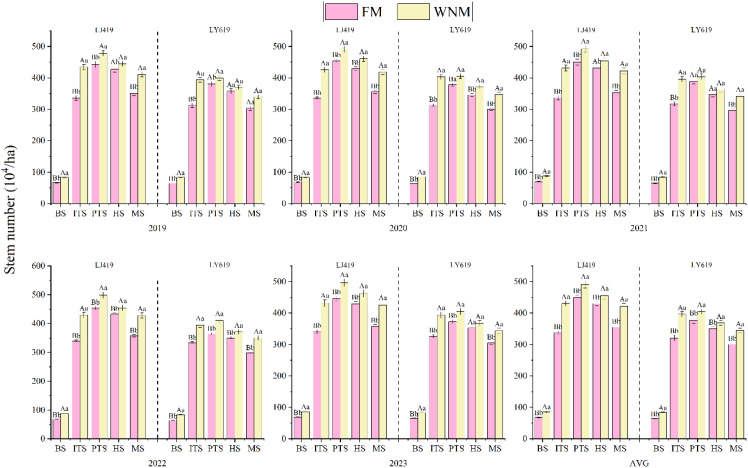
Tillering dynamics of representative growth stages. AVG is 2019–2023 average actual yield values. BS is basic seedlings stage, ITS is initial tillering stage, PTS is peak tillering stage, HS is heading stage, MS is maturity stage. LJFM is Liaojing419 with traditional farmers’ equal row spacing method, LJWNM is Liaojing419 with wide-narrow row spacing method, LYFM is Tianlongyou619 with traditional farmers’ equal row spacing method, LYWNM is Tianlongyou619 with wide-narrow row spacing method. Values within a column marked with different uppercase letters and lowercase letters are significantly different at the 0.05 and 0.01 levels in same varieties and in one year.

The variation in the growth rate during the stem and tiller growth periods is an important factor in measuring the population structure of rice and affects the number and quality of productive panicles during the maturity period. According to the analysis of stem and tiller dynamics (**Figure 3**), under the two methods, the stems of the two cultivars first increased, then decreased, and finally stabilized at maturity with the progression of the growth period. Except during the transplanting period, at the other four growth stages, the number of tillers was greater under the WNM than under the FM. Compared with those under the FM, the use of the WNM increased the five-year average numbers of basic transplanted LJ419 and LY619 seedlings by 25.8% and 30.3%, respectively. The increase rate of the number of stems under the WNM was greater than that under the FM at the peak stage, and the decline rate under the WNM was lower than that under the FM at the full panicle mature stage, resulting in a greater panicle formation rate under the WNM than under the FM. The average tiller and panicle formation rates of the LJ419 cultivar under the FM and WNM over the 5-year period were 79.0% and 85.7%, respectively. The average tiller and panicle formation rates of the LY619 cultivar under the FM and WNM over the five-year period were 79.7% and 85.1%, respectively.

### 3.4 Uniformity of the panicle neck height during the harvest period

The panicle neck height is the minimum distance between the panicle and the ground, which determines the threshing effect of the harvester after plant cutting. The average panicle height and uniformity can be used as important indicators to determine whether the panicle has entered the threshing bin or whether it has dropped. The average neck height of the cultivars varied between years. Notably, the average neck heights of the LJ419 and LY619 cultivars ranged from 82.1 to 85.3 cm and from 82.7 to 87.6 cm, respectively. The uniformity of WNM was significantly greater under than the FM, with five-year average values ranging from 85.6% to 86.8% and from 80.9% to 81.8%, respectively. Notably, the difference between the two methods for the LJ419 cultivar was greater. The main reason for the low uniformity under the FM was the number of plants with panicle neck heights between 35 and 60 cm. These plants exhibited relatively high dispersion with a low median value, which affected the overall uniformity of the rice population.

### 3.5 Uniformity of the length of dropped panicles during the harvest period

**Figure 4:**
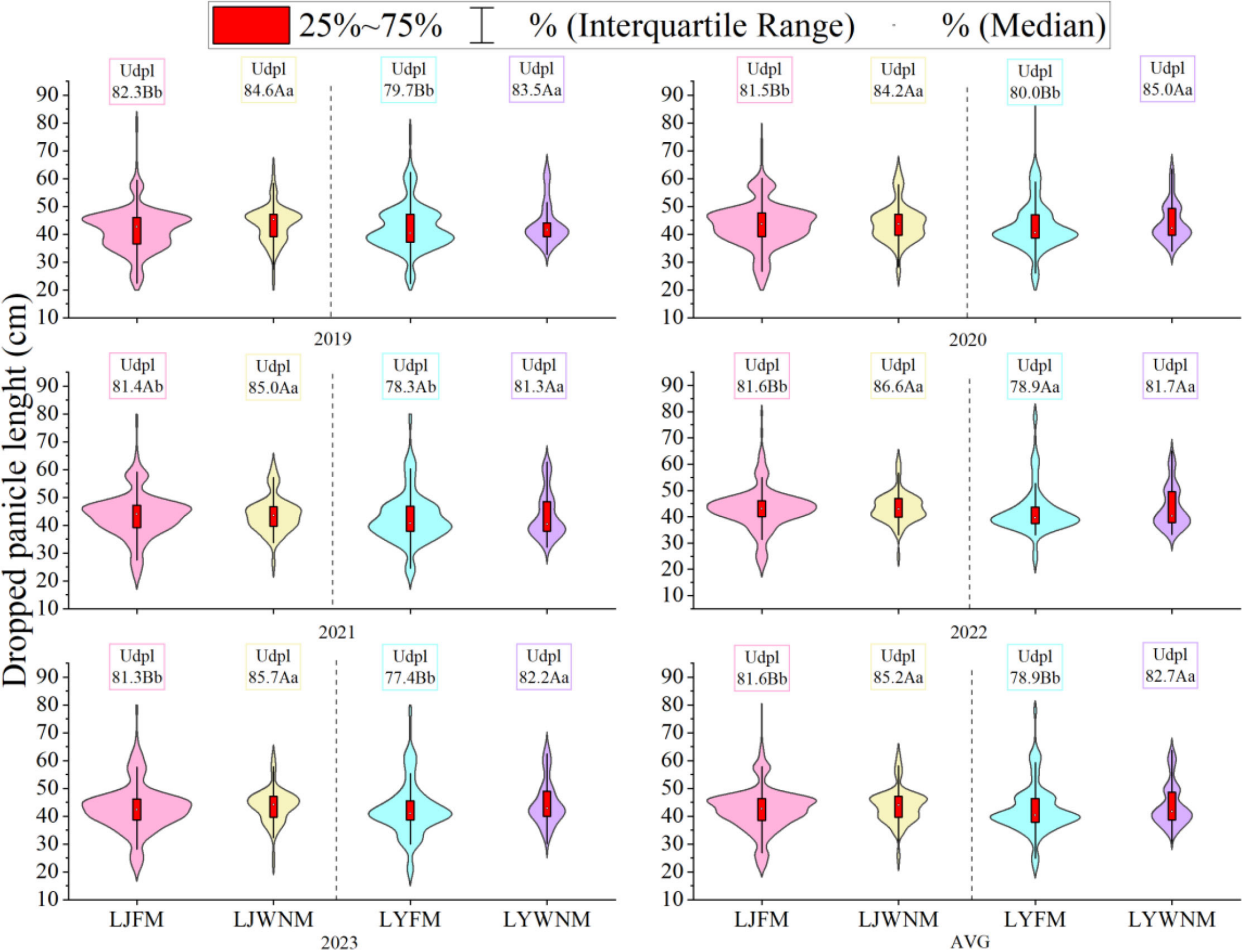
Dropped panicle lengths. AVGs are 2019–2023 average actual yield values. LJFM is Liaojing419 with traditional farmers’ equal row spacing method, LJWNM is Liaojing419 with wide-narrow row spacing method, LYFM is Tianlongyou619 with traditional farmers’ equal row spacing method, LYWNM is Tianlongyou619 with wide-narrow row spacing method. Udpl is uniformity of dropped panicle length. Values within a column marked with different uppercase letters and lowercase letters are significantly different at the 0.05 and 0.01 levels between different years, respectively.

After mechanized harvesting, dropped panicles are those that have not entered the threshing bin of the harvester, and the length and uniformity of dropped panicles provide the basis for assessing the occurrence of panicle dropping. The measurement and analysis of dropped panicles in the field during the harvest period (**Figure 4**) revealed that the average neck height of dropped panicles of a given cultivar varied between years. The lengths of dropped panicles ranged from 20~60 cm, and the average lengths for the LJ419 and LY619 cultivars ranged from 41.6~44.0 cm and 42.0~44.7 cm, respectively. The five-year average uniformity of the length of dropped panicles of a given cultivar was significantly greater under the WNM than under the FM. The uniformity values of the LJ419 cultivar were 81.6% and 85.2% under the FM and WNM, respectively, whereas those of the LY619 cultivar were 78.9% and 82.7% under the FM and WNM, respectively. The main source of this difference was the larger number of dropped panicles longer than 60 cm under the FM, whereas the median length of dropped panicles under the WNM was smaller. This was due to the more uniform development of stems and tillers under the WNM, which reduced the number of tillers at low panicle positions and increased the proportion of productive panicles.

### 3.6 Uniformity of the number of dropped panicles during the harvest period

**Figure 5:**
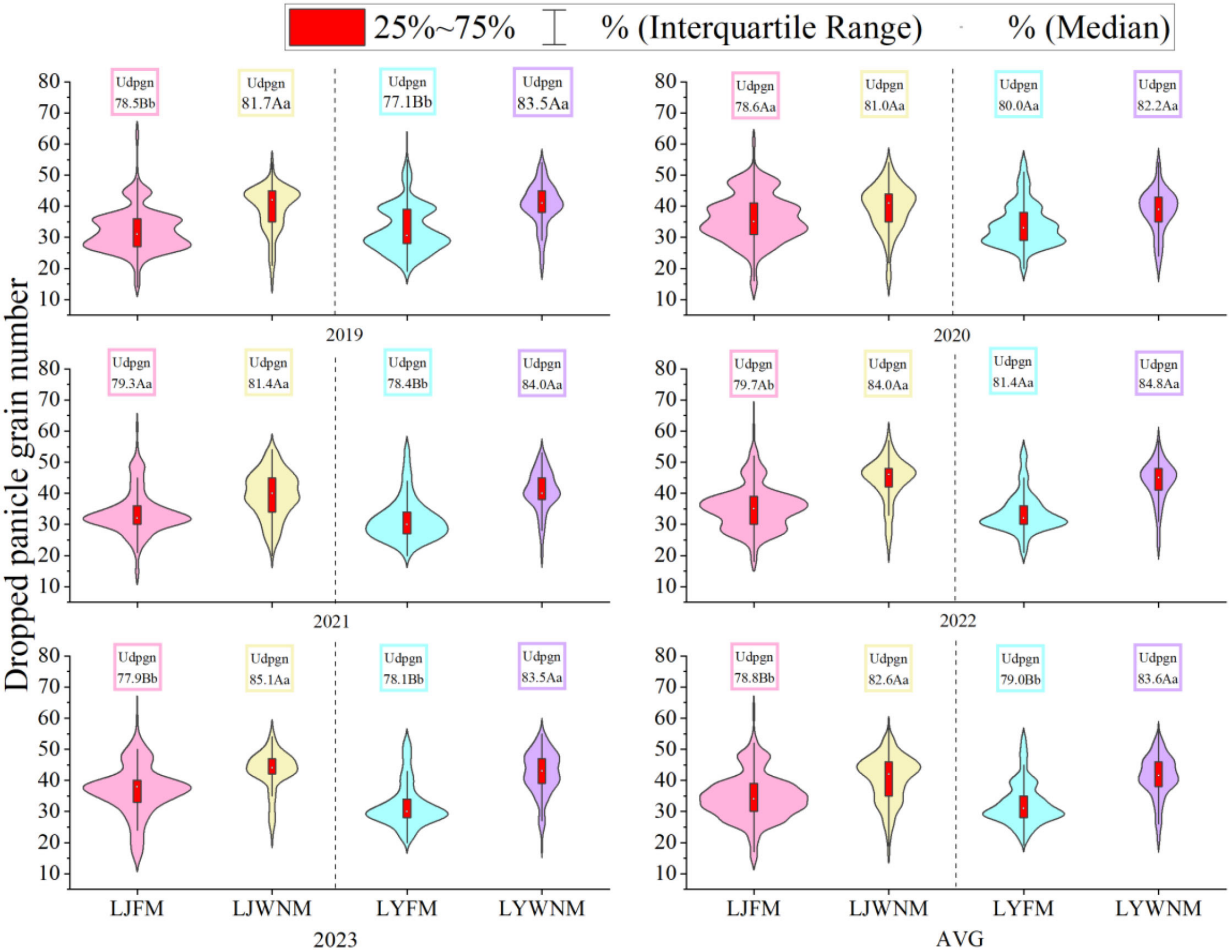
Dropped panicle grain numbers. AVGs are 2019–2023 average actual yield values. LJFM is Liaojing419 with traditional farmers’ equal row spacing method, LJWNM is Liaojing419 with wide-narrow row spacing method, LYFM is Tianlongyou619 with traditional farmers’ equal row spacing method, LYWNM is Tianlongyou619 with wide-narrow row spacing method. Udpgn is uniformity of dropped panicle grain number. Values within a column marked with different uppercase letters and lowercase letters are significantly different at the 0.05 and 0.01 levels between different years, respectively.

The DPGN during mechanized harvesting is an important indicator of yield loss and is influenced by the population and individual quality during the harvest period. The calculation and analysis of dropped panicles in the field during the harvest period (**Figure 5**) revealed that the interannual variation in the average panicle neck height of each cultivar was consistent. The DPGN values under both methods, except for extreme values, ranged from 20~55 grains per panicle. The FM exhibited the most instances within the range of 20~40 grains per panicle, whereas the WNM exhibited the most instances within the range of 35~45 grains per panicle. The five-year average uniformity of the number of dropped panicles of each cultivar was significantly greater under the WNM than under the FM. The proportions of the LJ419 cultivar were 78.8% and 82.6% under the FM and WNM, respectively, and the proportions of the LY619 cultivar were 79.0% and 83.6% under the FM and WNM, respectively.

### 3.7 Harvest loss and its composition

Mechanical losses during the harvest period affect the rice yield, and analyzing the constituent factors of these losses can provide insights for reducing losses. In this study, the number of dropped panicles varied significantly between cultivars and methods, there was no significant difference between years (**Table 2**). The average decrease in the number of panicles of the LJ419 cultivar over the five-year period was 54.1/m^2^ and 28.4/m^2^ under the FM and WNM, respectively, whereas for the LY619 cultivar, the value was 44.3/m^2^ and 20.0/m^2^ under the FM and WNM, respectively. The DPN, dropped panicle rate, DPGN, dropped panicle grain rate, and yield loss rate were significantly greater under the FM than under the WNM for the same cultivar. The 5-year average yield loss rates of the LJ419 cultivar under the FM and WNM were 4.4% and 2.4%, respectively, whereas those of the LY619 cultivar under the FM and WNM were 3.5% and 2.0%, respectively. The yield loss and yield loss rate increase with increasing yield in the same method with two cultivars. However, the WNM treatment resulted in a lower yield loss and the yield loss rate in the same cultivar.

**Table 2:** Harvest loss and its composition.

Y	M	DPN /m^2^	DPR %	DPGN /m^2^	DPGR %	DPGW g/m^2^	YLR %
2019	LJFM	56.7Aa	13.9Aa	1823.6Aa	3.8Aa	45.6Aa	4.1Aa
	LJWNM	29.4Bb	6.7Bb	1156.0Bb	2.3Bb	28.4Bb	2.5Bb
	LYFM	45.4Aa	12.3Aa	1477.4Aa	3.9Aa	35.6Aa	3.7Aa
	LYWNM	22.5Bb	6.2Bb	909.6Bb	2.4Bb	22.2Bb	2.3Bb
2020	LJFM	53.7Aa	13.1Aa	1930.5Aa	4.1Aa	48.4Aa	4.3Aa
	LJWNM	28.5Bb	6.4Bb	1125.2Bb	2.2Bb	27.4Bb	2.4Aa
	LYFM	44.1Aa	12.1Aa	1491.5Aa	4.0Aa	36.6Aa	3.9Aa
	LYWNM	20.1Bb	5.5Bb	777.5Bb	2.0Bb	19.3Bb	2.0Bb
2021	LJFM	51.5Aa	12.7Aa	1727.2Aa	3.6Aa	41.8Aa	3.7Aa
	LJWNM	28.8Bb	6.4Bb	1136.7Bb	2.3Bb	27.4Bb	2.3Bb
	LYFM	43.1Aa	12.0Aa	1357.8Aa	3.6Aa	34.3Aa	3.7Aa
	LYWNM	19.3Bb	5.3Bb	783.0Bb	2.0Bb	20.2Bb	2.1Bb
2022	LJFM	53.5Aa	13.0Aa	1875.4Aa	3.8Aa	47.2Aa	4.1Aa
	LJWNM	28.4Bb	6.2Bb	1254.4Bb	2.4Bb	31.4Bb	2.6Bb
	LYFM	45.9Aa	12.6Aa	1544.6Aa	4.0Aa	39.0Aa	4.0Aa
	LYWNM	19.0Bb	5.1Bb	830.1Bb	2.1Bb	21.3Bb	2.1Bb
2023	LJFM	55.1Aa	13.4Aa	2014.1Aa	4.2Aa	50.8Aa	4.4Aa
	LJWNM	26.8Bb	5.9Bb	1154.2Bb	2.2Bb	28.4Bb	2.4Bb
	LYFM	43.1Aa	11.7Aa	1377.9Aa	3.5Aa	34.7Aa	3.5Aa
	LYWNM	18.9Bb	5.2Bb	799.3Bb	2.0Bb	19.8Bb	2.0Bb
F-value	V	1440.68**	142.66**	116.79**	172.57**	3.35ns	17.63*
	M	2206.43**	430.17**	322.42**	3775.16**	568.08**	436.80**
	Y	0.00ns	1.49ns	1.52ns	0.00ns	1.62ns	1.05ns
	V×M	1.09ns	1.66ns	2.83ns	0.47ns	0.20ns	0.01ns
	V×Y	0.14ns	1.10ns	1.16ns	0.18ns	0.94ns	1.27ns
	M×Y	0.70ns	1.11ns	1.35ns	0.41ns	0.72ns	1.24ns
	V×M×Y	14.13**	25.46**	16.38**	19.31**	11.73**	13.40**

LJ, variety of LJ419; LY, variety of TLY619;.FM, traditional farmers’ equal row spacing method; WNM, wide-narrow row spacing method; Y, year; V, variety; M, method; ns, no significant difference; * and ** indicate significance at the 0.05 and 0.01 levels, respectively. DPN, dropped panicle number; DPR, dropped panicle rate; DPGN, dropped panicle grain number; DPGR, dropped panicle grain rate; DPGW, dropped panicle grain weight; YLR, yield loss rate. Values within a column followed by different uppercase letters and lowercase letters are significantly different at the 0.05 and 0.01 levels in same varieties and in one year.

**Figure 6:**
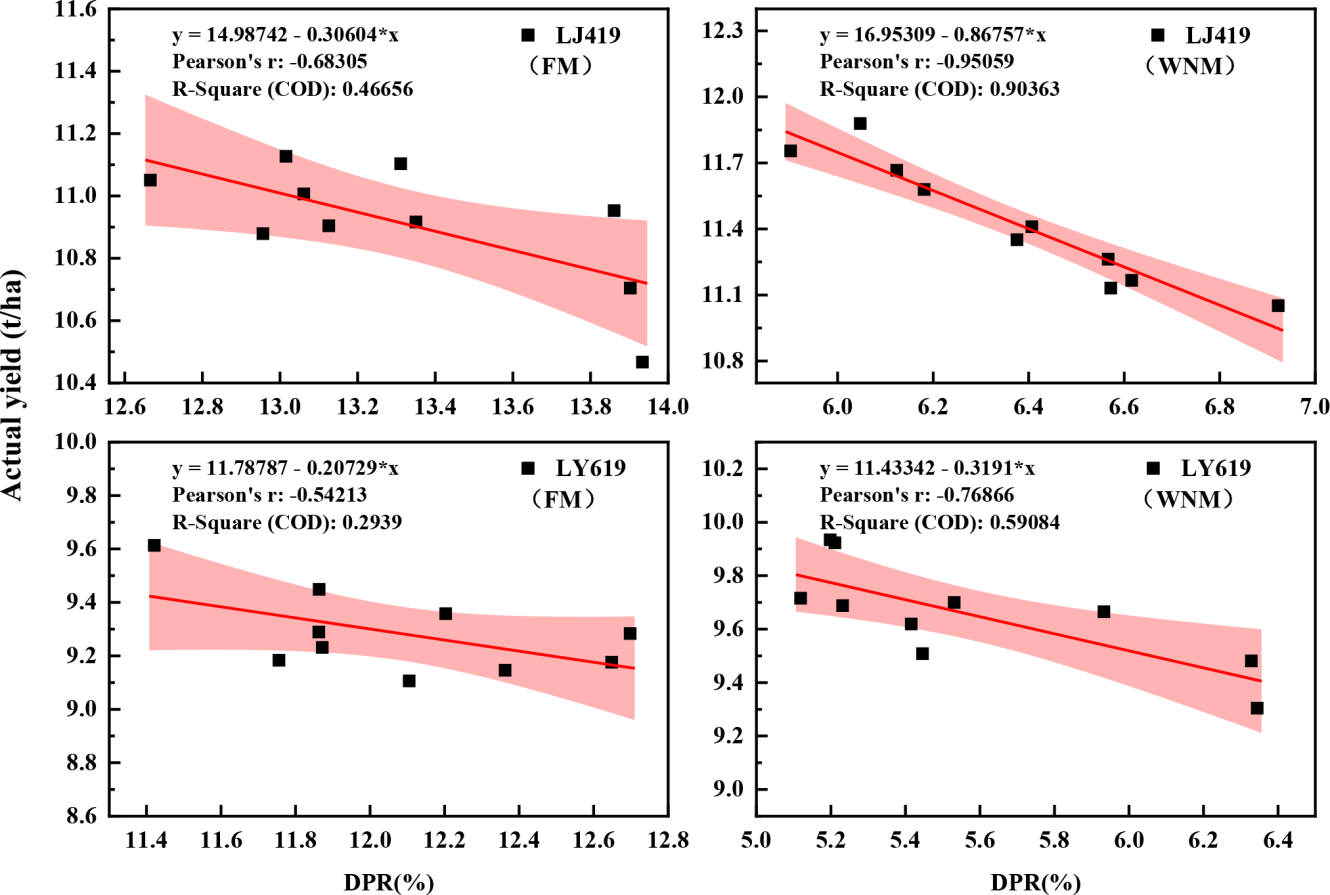
Relationship between the yield and the dropped panicle rate. DPR is dropped panicle rate, FM is traditional farmers’ equal row spacing method, WNM is wide-narrow row spacing method.

The proportion of dropped panicles is an important indicator for evaluating the quality of mechanized rice harvesting. The LJ419 and LY619 cultivars exhibited a liner negative correlation between the yield and the proportion of dropped panicles under both the FM and WNM (**Figure 6**), with R^2^ values of 0.46656, 0.90363, 0.2939, and 0.59084, respectively. The correlation was stronger under WNM than under FM, and the high−yield cultivar LJ419 generally exhibited higher yields than the good−tasting cultivar LY619. Notably, under WNM, the LJ419 cultivar, which achieved the greatest yield increase in this study, displayed a highly significant negative correlation between yield and the number of dropped panicles. This association, however, should be interpreted with caution, as it may partly arise from the mathematical relationship between final yield and its loss components, rather than a purely biological mechanism. Nonetheless, it could also reflect that high−yielding populations with greater plant uniformity at maturity tend to experience reduced mechanical harvest losses. Therefore, optimizing transplanting density and configuration to improve population uniformity may contribute to both higher yields and lower harvest losses, supporting stable and high rice production.

### 3.8 Analysis of the rice yield contribution

**Figure 7:**
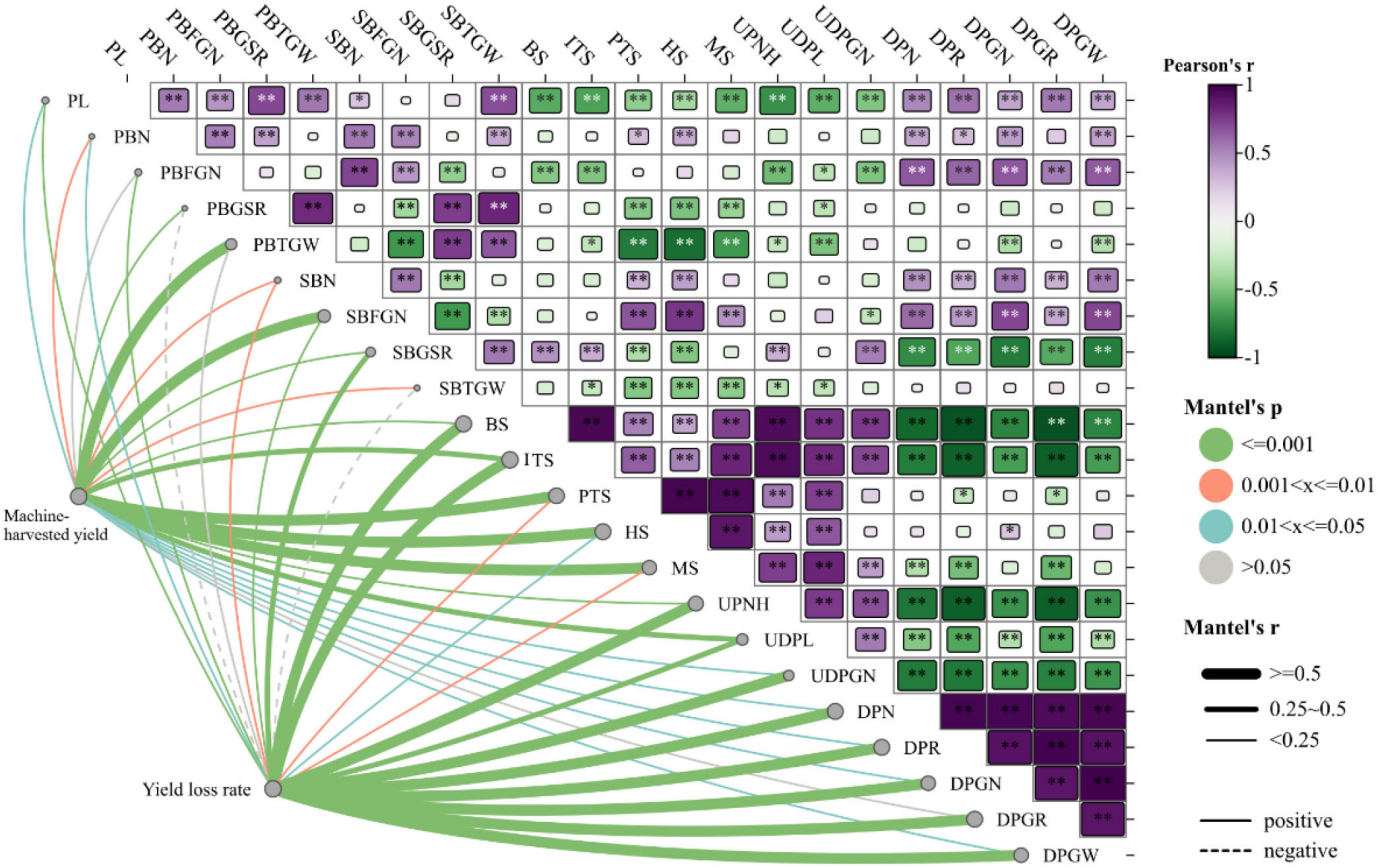
Mantel test for the correlation between various factors and rice yield and loss. PL is panicle length, PBN is primary branch number, PBGN is primary branch grain number, PBSSR is primary branch seed setting rate, PBTGW is primary branch thousand-grain weight, SBN is secondary branch number, SBGN is secondary branch grain number, SBSSR is secondary branch seed setting rate, and SBTGW is secondary branch thousand-grain weight, BS is stem number of basic seedlings stage, ITS is stem number of initial tillering stage, PTS is stem number of peak tillering stage, HS is stem number of heading stage, MS is productive panicle number of maturity stage, Upnh is uniformity of panicle neck height, Udpl is uniformity of dropped panicle length, DPN is dropped panicle number, Udpgn is uniformity of dropped panicle grain number, DPR is dropped panicle rate, DPGN is dropped panicle grain number, DPGR is dropped panicle grain rate, DPGW is dropped panicle grain weight. The solid line denotes that the impact is statistically significant, and the thicker the line, the greater the impact. The dotted line indicates that the impact is not statistically significant.

Reducing the mechanical yield loss on the basis of a higher harvest yield can increase the rice yield. In this study, the number and length of dropped panicles were significantly negatively correlated with the uniformity of the panicle neck height, the uniformity of the PL, and the uniformity of the number of dropped panicles (**Figure 7**). The uniformity of the panicle neck height was significantly positively correlated with the number of tillers during rice growth, especially the number of productive panicles. This finding indicates that increasing the number of tillers, stabilizing the uniformity of productive panicles at maturity, maintaining a high uniformity of the panicle neck height, and mechanically harvesting plants with uniform field population structure and position features are beneficial for increasing yields. In this study, there were varying degrees of negative correlations between the number of productive panicles at the mature stage and the other yield components except for the number of grains on secondary branches, thus indicating that multiple uniform panicles are beneficial for increasing yields.

## 4 Discussion

To better understand the response of rice agronomic traits to different methods during mechanized harvesting, we incorporated the plant morphology of two cultivars and two planting methods throughout five rice-growing seasons within a unified framework. We chose the fertilization method with the highest production benefits and the widest planting area. At the same time, our research is also aimed at practical production, solving the problems existing in the current farmers’ planting methods, and providing a basis for reducing mechanization losses and increasing rice yield under this method. We aimed to provide a holistic perspective on increasing yields and reducing losses in large-scale mechanized field trials. This approach differs from those in previous studies in which each factor was considered separately.

Differences in planting methods result in changes in the number of individuals and the population structure at different growth stages, leading to differences in the morphology of mature populations and changes in warehouse yields and dropped panicle losses under the same mechanized harvesting conditions (Li et al., 2010; Yin et al., 2016; Liu et al., 2020; Pan and Pan, 2022). Therefore, establishing a mature rice population structure to increase yields while reducing losses is particularly important for the harvest and storage yields of rice.

### 4.1 Changes in yield and panicle distribution

The uniformity of crops is closely related to population growth, and uneven rice growth is a common phenomenon (Liu et al., 2004; Huang et al., 2017). The differences in the growth rate and competition among individuals are the main reasons for a low population uniformity. By improving the spatial configuration of row hole spacings, increasing the number of productive panicles, and increasing the number of secondary branches and their uniformity, a population with a high capacity for machine-planted rice can be obtained. Moreover, a higher grain weight is beneficial for increasing the yield (Du et al., 2014). Our team’s previous research on the three densification methods (conventional densification 30 ×14cm, equal narrow row spacing densification 25×18cm, wide and narrow spacing densification 36 + 14 ×18) and FM showed that the conventional densification 30 ×14cm, equal narrow row spacing densification 25×18cm have a no significant difference in yield compared to FM, although they have the higher basic number of seedlings. While, WNM have a significant difference in yield compared to FM. Proving the effectiveness of optimizing row spacing configuration (WNM) in improving rice yield (Dong et al., 2025). In this study, the numbers of productive panicles of the LJ419 and LY619 cultivars increased by 18.7% and 14.7%, respectively, under the WNM compared with those under the FM (**Table 1**), which is the key to yield improvement. Moreover, increasing the density of wide and narrow rows can be employed as a cultivation measure for increasing yields.

Deng et al. (2023) and Deng et al. (2022) reported that the spatial structure configuration affects the canopy structure and the population photosynthetic efficiency, leading to changes in the rice canopy microecology as well as in the filling rate and filling capacity at the later rice growth stages. Xiong et al. (2021) reported that the high light efficiency under the WNM is due to establishment of ventilated and transparent corridors while changing flat light conditions into three-dimensional light conditions, thereby fully leveraging the marginal advantages of the populations, increasing the photosynthetically active radiation (PAR) interception rate in the middle and lower layers (especially in the middle layer), and maximizing the potential advantages of cultivar yields. In this study, the use of the WNM significantly reduced the thousand-grain weight of the primary and secondary branches of the LJ419 cultivar after densification, but the PPN, seed setting rate, and productive panicle rate (**Table 1**) were greater, with more complete filling and the highest grain-filling rate, resulting in an overall increase in the yield. After densification via the WNM, LY619 maintained a relatively large number and high quality of panicle grains while increasing the number of productive panicles, thereby increasing the rice harvest yield.

The panicle uniformity, productive tiller ratio and productive tiller number largely affects tiller and panicle formation, the production and accumulation of photosynthetic substances, and the yield composition (Zheng et al., 2021; Liu et al., 2022). In this study, the configuration of the LJ419 cultivar with alternating wide–narrow row spacings increased the number of productive panicles more significantly (**Figure 1**), with the greatest yield improvement (5-year average of 5.54%). The LY619 cultivar demonstrated improvements in the number of productive panicles, resulting in an increase in the harvested rice yield (5-year average of 4.49%). In summary, the application of the WNM is more conducive to maximizing the growth advantages and increasing the potential yield of different types of machine-inserted rice than the application of the FM. An appropriate row spacing configuration can effectively increase the rice yield.

### 4.2 Changes in the mechanical harvesting of dropped panicle parts

**Figure 8:**
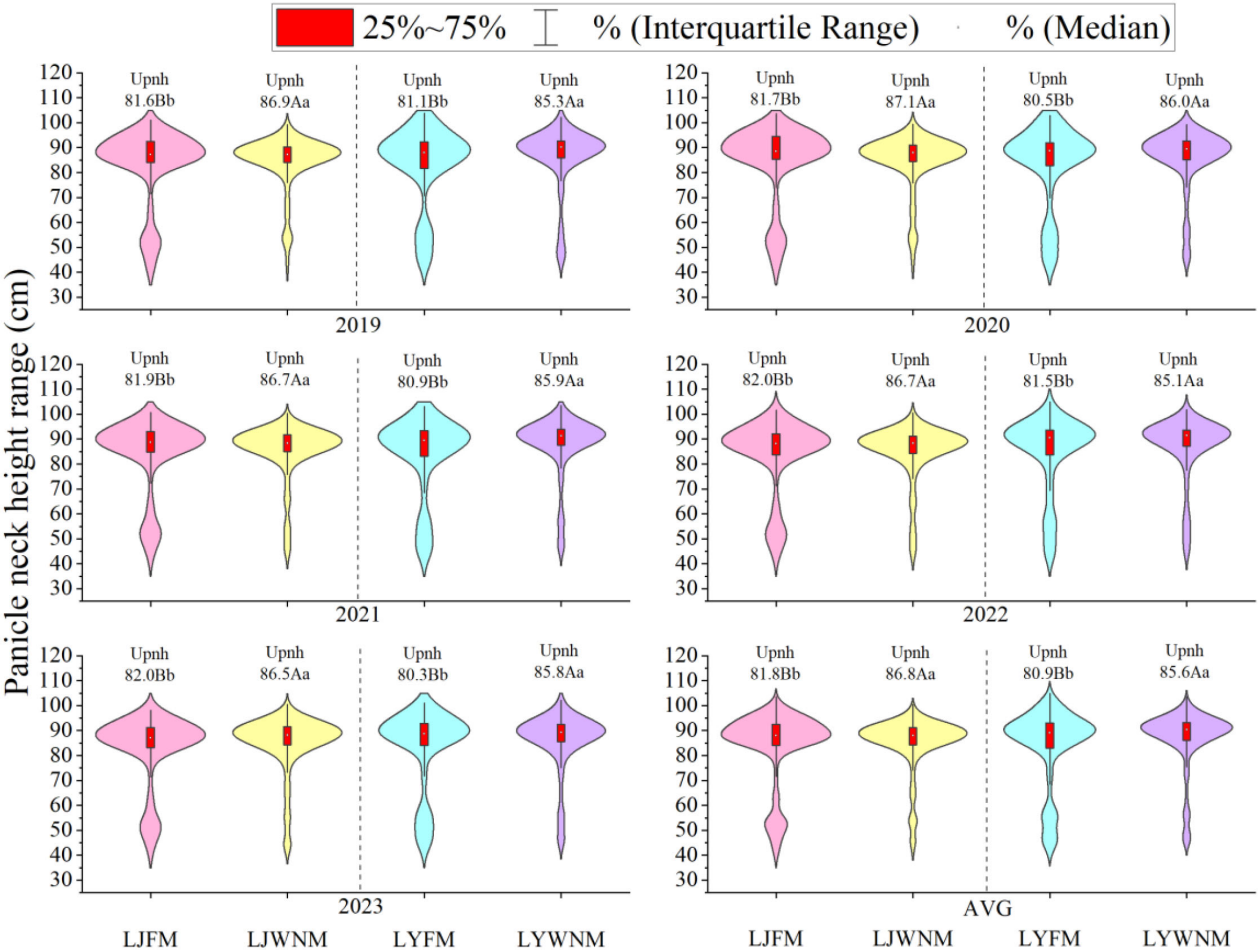
Panicle neck height ranges. AVGs are 2019–2023 average actual yield values. LJFM is Liaojing419 with traditional farmers’ equal row spacing method, LJWNM is Liaojing419 with wide-narrow row spacing method, LYFM is Tianlongyou619 with traditional farmers’ equal row spacing cultivation method, LYWNM is Tianlongyou619 with wide-narrow row spacing method. Upnh is uniformity of panicle neck height. Values within a column marked with different uppercase letters and lowercase letters are significantly different at the 0.05 and 0.01 levels between different years, respectively.

The yield loss during the rice harvest indicates that all the production inputs generated by rice before the loss are meaningless and constitute complete waste (Zhao et al., 2023). Improving agronomic measures and developing a planting method that facilitates easy mechanized harvesting can be an important way to improve quality and reduce damage during rice machine harvesting. Yu et al. (2021) reported that improving cultivation measures can increase the panicle position uniformity, reduce the lodging rate, effectively alleviate the negative impact of densification, and decrease the yield loss rate. The wide–narrow row planting method for inserted rice produces high-quality rice with a uniform distribution in the field, and the individual production capacity and group advantages are fully utilized. The population at maturity is uniform, which can improve the quality of the machine-inserted rice population and increase the rice yield (Dong et al., 2023). In this study, the early tiller of FM grows normally. However, in the later stage, due to individual material accumulation issues, the plants become shorter and form a dispersed pattern of plant height within the population, causing a decrease in uniformity of the panic neck height (**Figure 3**). There was a slight difference in the average neck height between the two cultivars at the mature stage, whereas the uniformity of the panicle neck height at the mature stage under the FM was significantly lower than that under the WNM, resulting in uneven panicle positions and suggesting an association with the inability to better match the cutting operation of the harvester, which may contribute to increased panicle dropping. Owing to the larger number of stems and tillers and greater dispersion, the LJ419 cultivar exhibited lower uniformity in terms of the panicle neck height and a corresponding larger decrease in the panicle number (**Figure 8**).

When rice is harvested, the stem cutting position is relatively high, causing some panicles with lower plant heights to occur out of reach of the harvesting platform and be missed, resulting in dropped panicles, i.e., dry matter loss (Pan and Pan, 2022; Xu et al., 2023). Wang et al. (2016) studied the composition of rice harvest losses in a rice–wheat rotation system at the mature stage and reported that the main source of the total losses was dry matter loss. Therefore, by evaluating the characteristics of missed harvests, the proportion of relevant traits can be reduced to increase grain return and rice yield levels. Zeng et al. (2014) proposed a suitable stubble height of 10 cm for machine harvesting, which is suitable for early rice cultivars with small plant heights. Xu et al. (2023) studied double-season hybrid rice and suggested that the greater the distance from the stem cutting point to the top of the panicle is, the greater the operating load and the higher the wind force needed. As a result, more grains are discharged through the air outlet, and the stem cutting area is too small, resulting in some panicles with smaller heights not reaching the harvesting platform and being missed. In terms of production, especially for semifed harvesting models, the losses can be greater. In this study, the ratio of the number of dropped panicles during mechanized harvesting to the number of stems harvested at the full heading and mature stages was consistent, suggesting that whether the stem tillers became productive panicles (in line with the height of the machine header) basically determined their ability to be mechanically harvested (**Figure 8**). Therefore, it can be inferred that the productive panicle rate at the mature stage can be optimized by controlling the panicle neck height during the harvest period, which ultimately affects the number and quality of dropped panicles (**Figure 5**, **Table 2**). Our finding that >75 cm panicle height is critical for harvest efficiency (**Figure 4**: dropped panicles ≤60 cm + 15 cm stubble height) aligns with mechanization principles but reveals key innovations. WNM’s 36 + 14 cm configuration increased panicle uniformity of panicles >75 cm. We infer that the wider rows (36 cm) may provide better header access, which could explain why WNM achieved lower loss than equal spacing at identical panicle height ranges. However, we acknowledge that this interpretation is based on observed patterns rather than direct measurements of header–plant interaction.

### 4.3 Integration of mechanized harvesting and the mature population structure

The in-depth integration of agricultural machinery and agronomy is an inevitable choice for comprehensively achieving the goals and development direction of agricultural mechanization and modernization (Gao et al., 2020). At present, mechanical harvesting losses include header losses, grain leakage losses, entrainment losses, and cleaning losses, making it difficult to achieve particle return to the warehouse; moreover, header losses are the most significant losses and are affected by the plant variety, planting method, and mechanical harvesting quality (Qiu et al., 2021; Pan and Pan, 2022). Within the context of large-scale mechanized rice harvesting, reducing losses and increasing yields can be achieved by matching agricultural machinery and adapting agricultural machinery to agronomy (Mu and Wu, 2023). In this study, the WNM met the height requirements of the parallel advancement process of the harvester by increasing the PPN and panicle uniformity during the maturity period, which was associated with a reducing the incidence of dropped panicles and the yield loss. In addition, this study revealed that, compared with the use of equal row spacings (30 cm) under the FM, the use of wide row spacings (36 cm) under the WNM provides large corridors for the cutting platform to pass through (172 cm (FM: 5 rows; WNM: 6 rows)), which we infer enables sufficient contact during the harvesting operation while avoiding adjacent row damage and reducing the likelihood of accidental collision with normal plants causing panicle dropping. This interpretation, however, is based on spatial geometry rather than direct measurements of mechanical contact frequency. Therefore, there were fewer abnormal dropped panicle values under WNM, suggesting that this method may better facilitate longitudinal cutting by the harvester’s cutting table.

There is a close relationship between the moisture status of rice plants during harvesting, the height of harvested plants, the position of panicles entering the threshing bin, the ventilation intensity, and the rate of machine-harvested rice loss (Doungpueng et al., 2020; Wang et al., 2021). The harvest time in this study was 10:00 am on the second sunny day after autumn frost. The moisture content in the rice grains was consistent, and all harvesters were of the same model with consistent mechanical harvesting parameters. Therefore, it is necessary to consider the impacts of the harvested plant height and the position of panicles entering the threshing bin on the yield loss. The number of panicle neck height > 75cm was higher under the WNM (**Figure 8**), which may allow the panicle to enter the threshing bin in a relatively concentrated position after passing through the cutting table, thus potentially reducing the incidence of smaller panicles being excluded owing to their mixing with plant stems and not entering the threshing bin. This may have further contributed to reduced losses in the threshing bin and better achievement of agronomic–mechanical integration.

### 4.4 Limitations

While increasing rice yields and reducing losses in the field has been extensively studied worldwide, research on the response of rice agronomic traits to various methods during mechanized harvesting processes remains limited (Zhao et al., 2023; Hu et al., 2020). The advantage of WNM lies in spatial heterogeneity rather than density, and the 25cm equal spacing fails due to the inability to overcome the physiological mechanical contradiction. Although seedling density was standardized, the interaction between row spacing and density thresholds requires deeper exploration. Furthermore, the small sample sizes for some variables may lead to nonsignificant or biased results (Yin et al., 2016; Pan and Pan, 2022). For example, the impact of different harvester models and header operation methods remains unknown due to the lack of observations. Additionally, the grains dropped during harvesting operations are important and cannot be ignored (Dong et al., 2023; Li et al., 2023; Wang et al., 2022; Zhao et al., 2023). Notably, the rice quality of the two cultivars was not included in our research since our dataset focused mainly on the changes in panicles and population uniformity at maturity (**Figures 3**, **8**, respectively). It is important to note that the two rice cultivars used in this study were managed under their respective locally recommended nitrogen fertilization regimes: the high−yield cultivar LJ419 received 300 kg N ha^-^¹, while the high−quality cultivar LY619 received 150 kg N ha^-^¹. Consequently, any observed differences in yield, yield components, tillering dynamics, and harvest losses between cultivars cannot be attributed solely to genetic factors, as they are confounded by the distinct nitrogen levels. The superior performance of LJ419 in terms of yield may therefore be partially driven by the higher nitrogen input, which promotes tiller production and biomass accumulation. In contrast, the lower nitrogen rate for LY619 aligns with practices aimed at enhancing grain quality, but may limit its tillering capacity and affect panicle uniformity. This management−specific confounding means that conclusions regarding cultivar superiority are strictly conditional on the associated agronomic regimes. Future studies should aim to disentangle genetic from management effects by testing multiple cultivars under a common nitrogen platform, or by including nitrogen as an additional experimental factor.

A key limitation of the present study is the indirect nature of the evidence supporting the proposed mechanisms underlying WNM’s effects. We did not conduct direct measurements of light distribution within the canopy, photosynthetically active radiation (PAR) interception, or detailed canopy structural parameters that could mechanistically link row spacing configuration to improved photosynthetic performance. Similarly, our conclusions regarding the role of “corridor space” in reducing header contact and panicle dropping are inferential, based on observed differences in panicle height uniformity, spatial geometry, and final loss outcomes, rather than on direct measurements of mechanical contact frequency or header–plant interaction dynamics. Therefore, while the observed patterns are consistent with our proposed explanations, the underlying mechanisms should be interpreted with appropriate caution.

Future research should aim to validate these mechanistic inferences using more direct approaches, such as canopy imaging and three−dimensional reconstruction to quantify light distribution and canopy architecture, sensor−based measurements of PAR interception across different row configurations, and high−speed video analysis or instrumented harvesters to directly quantify mechanical contact frequency and header–plant interaction patterns. Such studies would provide the direct evidence needed to confirm the mechanistic pathways proposed here.

Different transplanter models were used for FM (Kubota) and WNM (Jiufu) treatments, as required by their distinct row spacing patterns. Both machines were equipped with identical navigation systems and operated by experienced technicians to ensure consistent transplanting quality. However, potential performance differences between machines may still exist, and we acknowledge this as a study limitation. Future research should consider using a single adjustable transplanter or conduct pre-trial calibration tests to verify machine equivalence. Thus, future studies should focus on the comprehensive analysis of more production factors to provide more meaningful analysis results. In addition to increasing rice yields and reducing losses, the response of rice plants and panicles to the climate at maturity must be considered for complete and accurate harvest assessments. The application of wide–narrow row planting transplanter may increase inputs (Li et al., 2023; Zhu et al., 2018; Hu et al., 2018; Yang et al., 2014), and the number of transplanting seedlings and water and fertilizer management must be explored further. Such investigations could help meet the continuous demand for food to increase rice yields. However, the dynamic increases in the rice yield and rice quality resulting from WNM-based transplanting in paddy fields require further elucidation. Future work must quantify cost–benefit of WNM transplanter, test header adaptations (e.g., adjustable tine angles for wide rows), expand to diverse ecoregions.

## 5 Conclusion

The use of the WNM increased the rice yield by increasing the number of productive panicles, with the high-yield cultivar LJ419 showing a more significant improvement, with an average yield increase rate of 5.5%. By optimizing the number and quality of populations and individuals at different growth stages, the uniformity of the panicle neck height increased by 4.7~5.0%, ensuring the occurrence of panicles longer than 75 cm and reducing the number of dropped panicles.

Our study has important implications for addressing the dual challenges of increasing rice yields to meet the demand and reducing yield losses. Our work suggested that the WNM can be employed as a cultivation measure for increasing rice yields and reducing losses in mechanized production areas.

## Data Availability

The raw data supporting the conclusions of this article will be made available by the authors, without undue reservation.
