# Supplementary figures and images for: Wide-narrow row spacing reduces mechanical harvest losses in rice by optimizing panicle distribution: evidence from five-year field trials integrating density and cultivar variables
Source: Front Plant Sci. 2026 Apr 17;17:1796989. doi: 10.3389/fpls.2026.1796989 (PMC13133711; doi:10.3389/fpls.2026.1796989)

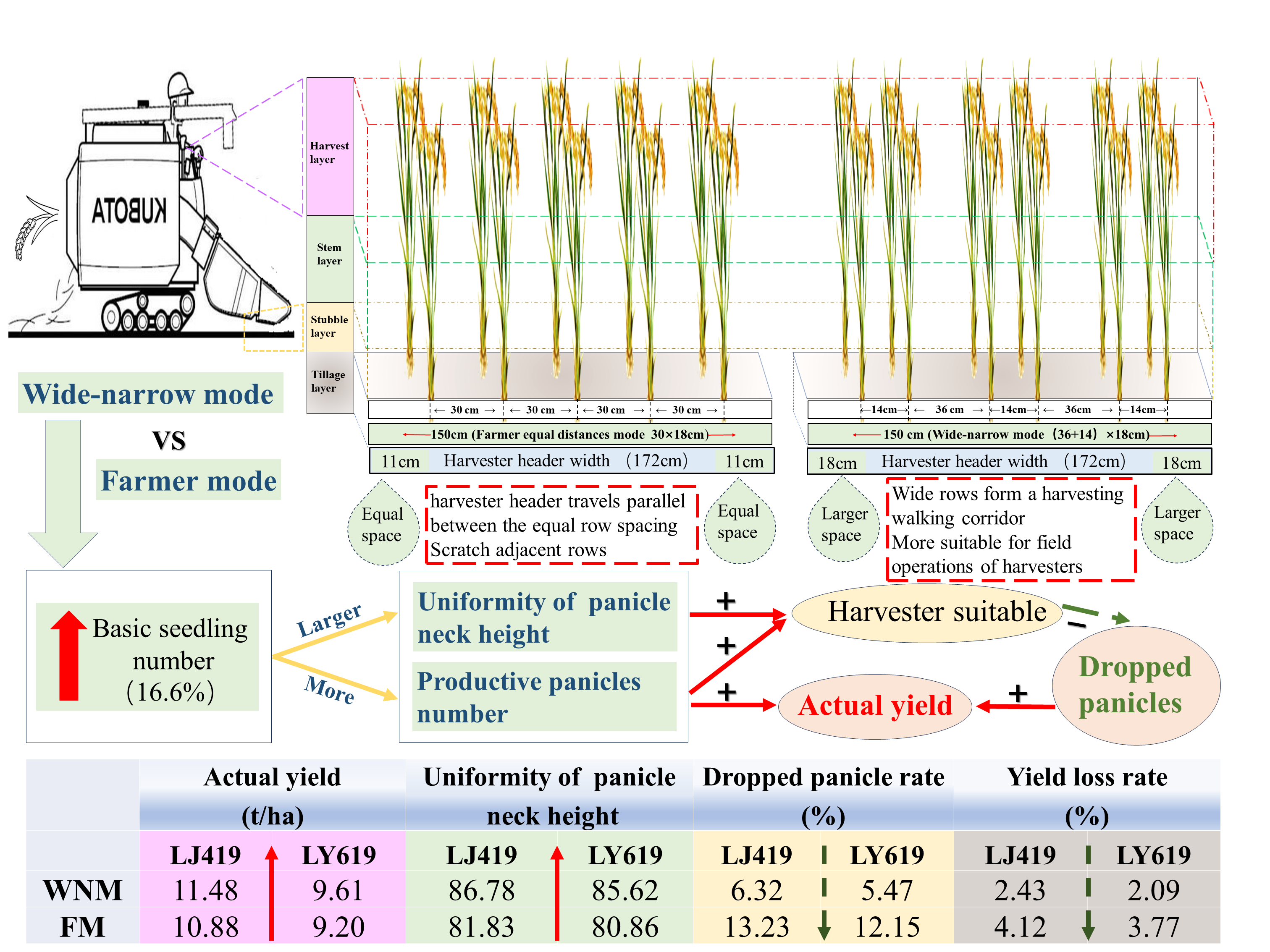

Supplement: Supplementary file 1 [file Image1.tif]
